# Coinfection of fungi with SARS-CoV-2 is a detrimental health risk for COVID-19 patients

**DOI:** 10.1186/s43088-022-00245-9

**Published:** 2022-05-04

**Authors:** Nahida Baten, Shah Wajed, Asma Talukder, Md. Habib Ullah Masum, Md. Mijanur Rahman

**Affiliations:** 1 Department of Microbiology, Noakhali Science and Technology University, Noakhali, 3814 Bangladesh; 2 Department of Biotechnology and Genetic Engineering, Noakhali Science and Technology University, Noakhali, 3814 Bangladesh

**Keywords:** COVID-19, SARS-CoV-2, Fungal coinfection, Mucormycosis, IPA, CAPA

## Abstract

**Background:**

Notable fungal coinfections with SARS-CoV-2 in COVID-19 patients have been reported worldwide in an alarming way. *Mucor* spp. and *Rhizopus* spp. were commonly known as black fungi, whereas *Aspergillus* spp. and *Candida* spp. were designated as white fungi implicated in those infections. In this review, we focused on the global outbreaks of fungal coinfection with SARS-CoV-2, the role of the human immune system, and a detailed understanding of those fungi to delineate the contribution of such coinfections in deteriorating the health conditions of COVID-19 patients based on current knowledge.

**Main body:**

Impaired CD4 + T cell response due to SARS-CoV-2 infection creates an opportunity for fungi to take over the host cells and, consequently, cause severe fungal coinfections, including candidiasis and candidemia, mucormycosis, invasive pulmonary aspergillosis (IPA), and COVID-19-associated pulmonary aspergillosis (CAPA). Among them, mucormycosis and CAPA have been reported with a mortality rate of 66% in India and 60% in Colombia. Moreover, IPA has been reported in Belgium, Netherlands, France, and Germany with a morbidity rate of 20.6%, 19.6%, 33.3%, and 26%, respectively. Several antifungal drugs have been applied to combat fungal coinfection in COVID-19 patients, including Voriconazole, Isavuconazole, and Echinocandins.

**Conclusion:**

SARS-CoV-2 deteriorates the immune system so that several fungi could take that opportunity and cause life-threatening health situations. To reduce the mortality and morbidity of fungal coinfections, it needs immunity boosting, proper hygiene and sanitation, and appropriate medication based on the diagnosis.

## Background

Severe acute respiratory syndrome coronavirus-2 (SARS-CoV-2) causes coronavirus disease 2019 (COVID-19), which is spread by human-to-human close contact, especially through respiratory droplets [1]. COVID-19 is a flu-like disease, bearing no symptoms in most infected individuals, but may develop signs and cause acute respiratory distress syndrome (ARDS), pneumonia, and even death [2]. Moreover, it is not only limited to respiratory illness but also has consequences for renal, hematological, and central nervous system (CNS) and develops a severe disease in older individuals and those with underlying medical conditions, including obesity [3], hypertension [4], rheumatic diseases [5], and diabetes mellitus [6, 7]. The intensity of mutation in spike proteins results in more powerful variants of SARS-CoV-2 such as B.1.1.7 (Alpha), B.1.351 (Beta), P.1 (Gamma), B.1.617.2 (Delta) [8], B.1.621 (Mu) [9], and B.1.1.529 (Omicron) [10] which could weaken the human immune system robustly. According to a retrospective cohort study, the individuals infected with alpha, beta, gamma, and delta variants have an elevated hospitalization risk compared to those infected with progenitor SARS-CoV-2 variants [11]. Because of prolonged hospitalization, the weakened immune system unleashes pathogens, mainly opportunistic fungi, which leads to the impairment of organs and even death [12].

However, there are tens of thousands of recognized fungi in nature, and among them, over 300 fungal species have been identified as human pathogens. Most fungal infections are caused by opportunistic fungi such as *Aspergillus, Candida, Cryptococcus,* and *Pneumocystis* [13]. In the case of COVID-19, patients with ARDS, hospitalized in intensive care units (ICU), receiving broad-spectrum antibiotics, going through invasive or noninvasive ventilation, and undergoing immunosuppressive or corticosteroid therapies are at the highest risk of getting opportunistic fungal infections [14]. Fungi responsible for these emerging coinfections, including *Mucor* spp. and *Rhizopus* spp., are named black fungi, whereas *Aspergillus* spp. and *Candida* spp. are called white fungi [15].

Furthermore, such fungal attacks caused reducing the number of CD4 + and CD8 + T cells resulting in disruption of the adaptive immune system in individuals infected with SARS-CoV-2 [12]. Basically, fungi are destroyed by CD4 + T cell-mediated adaptive immune responses, which protect cells from fungal attack through the action of IFN-γ from T helper cell 1 (Th1) or Interleukin-17 (IL-17) from Th17 cell. As the SARS-CoV-2 infected individuals have disrupted adaptive immune responses, the fungal infection takes over without any interference [16]. Moreover, the innate immune system also gets hampered by the “cytokine storm” due to ARDS and fails to give protection against the fungal pathogen [17].

Given the emphasis on the detrimental effects of fungal coinfections with SARS-CoV-2 in COVID-19 patients, this review study gathers facts and findings to delineate the worldwide notable fungal coinfections; roles of the immune system in the infections; morphological features, pathogenesis, clinical results, and laboratory diagnosis; and control and prevention of those fungi to deliver a comprehensive overview.

## Main text

### Fungi involved in creating coinfection with SARS-CoV-2

Several fungal diseases have been documented with SARS-CoV-2 infections, including mucormycosis, COVID-19-associated invasive pulmonary aspergillosis (CAPA), invasive candidiasis, and pneumocystis pneumonia [18–21]. The etiologic agents of mucormycosis are *Rhizopus arrhizus*, *Rhizomucor pusillus*, *Apophysomyces variabilis*, and *Lichtheimia corymbifera* [22], whereas *Aspergillus fumigatus* and *Aspergillus flavus* were predominant in CAPA [18, 23–25]. Besides, several *Candida* spp. such as *C. albicans*, *C. tropicalis*, and *C. parapsilosis* have been reported in invasive candidiasis [18]. Contrarily, pneumocystis pneumonia caused by *Pneumocystis jirovecii* has been documented in rare occurrences [18, 26]. Understanding the structure, pathogenicity, clinical sign symptoms, and laboratory diagnosis of those fungi would be helpful to outline their contribution to worsening the health conditions of COVID-19 patients (Table 1). There are many symptoms shared by *Mucor* and *Rhizopus* infections, such as chest pain, dyspnea, fever, headaches, tiredness, coughing, blisters on the skin, and a stomachache. The diagnosis varies, except for the similarity in a computed tomography scan’s result [27]. The morphologic features of *Mucor* and *Rhizopus* are also similar in several characteristics. They are saprophytic colonizers, filamentous, and have a stiff cell wall but vary in possessing sporangiospores and a stolon [28]. Also, *Aspergillus* and *Candida* have almost identical morphological features but distinct pathways for causing illness. *Aspergillus spp.* infects respiratory and nasal tissues, whereas *Candida spp.* attacks mainly endothelium and epithelial cells. The symptoms are significantly different in this situation because *Aspergillus spp.* has the most substantial match with the SARS-CoV-2 pathways and remarkably impacted the health of COVID-19 patients [29, 30].

**Table 1:** Morphological features, pathogenicity, clinical findings, and laboratory diagnosis of the fungi causing coinfection with SARS-CoV-2

Name of fungi		Morphology	Pathogenicity	Clinical manifestation	Symptoms	Diagnosis	References
Black Fungi	*Mucor* spp.	1. Saprophytic colonizers	Infection is assumed to spread by	Involved in creating infections to immunocompromised patients such as	Mucormycosis symptoms are mild and nonspecific, such as	Diagnosis is performed by	[56]
		2. Encompasses filamentous mycelium or budding yeast cells that are spherical	1. Inhalation, traumatic inoculation or ingestion	1. Pulmonary mucormycosis	1. Chest discomfort	1. Calcofluor white	
		3. Contain branched sporangiospores	2. Invasion of blood vessels, which results in tissue infarction, necrosis, and thrombosis	2. Rhinocerebral mucormycosis	2. Dyspnea	2. Fluorescent in situ hybridization	
		4. Contain rigid cell walls with the presence of cellulose or chitin		3. Subcutaneous mucormycosis	3. Fever	3. Gomori methenamine silver stain	
		5. Cell wall consists of lipids, proteins, phosphates, amino sugars, Phosphorus, Magnesium, and Calcium		4. Maxillofacial mucormycosis	4. Headache	4. Immunohistochemistry analysis	
				5. Gastrointestinal mucormycosis	5. Fatigue	5. Periodic acid–Schiff stain	
					6. Cough	6. Wet mount	
					7. Mucosal necrosis	7. Conventional PCR	
					8. Ophthalmologic abnormalities such as proptosis, ptosis, aphasia, and visual alterations	8. DNA sequencing	
					9. Nasal bridge or upper inside of black mouth lesions that rapidly worsen	9. Real-time PCR	
					10. Breathing problems	10. Restriction fragment length polymorphism	
					11. Infected skin might develop blisters or ulcers, and the region may turn black	11. API ID32C and API ID50C	
					12. Discomfort, warmth or redness or swelling surrounding the affected area	12. ELIspot	
					13. Bleeding in the digestive tract	13. Computed tomography (CT) scan	
					14. Stomachache		
	*Rhizopus* spp*.*	Differ with *Mucor spp.* in having unbranched sporangiospores and having stolon	Infection is assumed to spread by	Involved in creating infections to immunocompromised patients such as	*Rhizopus* spp. also cause *Mucormycosis*; thus, the symptoms are the same	Diagnosis is carried out by-	[57, 58]
			1. Inhalation, traumatic inoculation or ingestion	1. Pulmonary mucormycosis	1. Chest discomfort	1. Computed tomography (CT) scan	
			2. Invasion of blood vessels, which results in tissue infarction, necrosis, and thrombosis	2. Rhinocerebral mucormycosis	2. Dyspnea		
				3. Subcutaneous mucormycosis	3. Fever		
				4. Maxillofacial mucormycosis	4. Headache		
				5. Gastrointestinal mucormycosis	5. Fatigue		
					6. Cough		
					7. Skin blisters		
					8. Stomach pain		
White Fungi	*Aspergillus* spp.	1. Appear in velvety yellow to green or blue or brown mold	Infection routes are	Clinical significances are	Clinical signs and symptoms are	Diagnostic procedures are	[59]
		2. Comprise conidiophores that could be lengthy, rough, pitted, or spiny	1. Respiratory route	1. Chronic cavitary pulmonary aspergillosis and aspergilloma	1. Anorexia	1. Wet mount	
		3. Conidiophores are either uniseriate or biseriate	2. In tissue where hyphal growth forms	2. Allergic bronchopulmonary aspergillosis	2. Weight loss	2. Gomori’s methenamine silver stain (GMS)	
		4. Conidia are globose or subglobose, thorny and size varies from 3.5 to 4.5 µm in diameter	3. Dissemination in extrapulmonary tissues	3. Allergic fungal sinusitis	3. Malaise	3. Periodic acid–Schiff (PAS)	
		5. Produces toxins	4. Paranasal sinuses	4. Rhinosinusitis	4. Sweating	4. Galactomannan (GM) detection in fluids	
			5. Fungal colonization in the gastrointestinal tract at the sites of the cornea	5. Cutaneous infection	5. Fever	5. Early bronchoalveolar lavage (BAL)	
				6. Central nervous system infection	6. Persistent productive cough	6. CT scan	
					7. Dyspnea	7. Thin-section chest computed tomography	
					8. Chest pain	8. Multidetector computed tomography (MDCT)	
					9. Rare occasional hemoptysis	9. Multislice spiral computed tomography (MSCT)	
					10. Pain in the face	10. High resolution computed tomography	
					11. Erythema	11. Abdominal computed tomography	
					12. Development of eschar	12. Paranasal computed tomography or MRI of the central nervous system (CNS)	
					13. Infected and swollen eyelids	13. In vivo confocal microscopy (IVCM)	
					14. Irritation in the nose	14. Tomographic imaging probe	
					15. Consciousness loss	15. Two-photon microscopy (TPM)	
					16. A change in mental state	16. PCR	
					17. Hemiparesis	17. DNA sequencing	
					18. Convulsions	18. Image-based automatic hyphae detection	
						19. Double-sandwich (ds) ELISA	
	*Candida* spp*.*	1. Diploid	Causing candidiasis by	Clinical symptoms are	All candidiasis disease signs include	Diagnosis could be made by	[60]
		2. Acquire dimorphism characteristic	1. Adhering to epithelial cells	1. Vulvovaginal candidiasis	1. Discharge from the uterus	1. Wet Mount	
		3. Comprise filamentous hyphae	2. Forming colonization	2. Onychomycosis	2. Irritation in the vaginal region	2. PCR	
		4. Secrets toxin	3. Penetrating epithelia or invading hyphae	3. Candidemia	3. Burning sensation in the vagina	3. Nucleic acid amplification tests (NAATs)	
			4. Disseminating vascular tissue	4. Intra-abdominal candidiasis	4. Dyspareunia	4. Mass spectrometry	
			5. Colonizing endothelia	5. Peritonitis	5. Dysuria	5. 1,3-1β D glucan	
				6. Biliary candidiasis	6. White patches emerge that resemble curd in the mouth, throat, tongue, and gum linings	6. Mannan–antimannan	
				7. Candida endophthalmitis	7. White lesions on the retinal surface		
					8. Loss of vision, which may be gradual or occur suddenly		
					9. Edema of the retina or papillary		
					10. Inflammation and stricture development in both intrahepatic and extrahepatic biliary systems		
					11. Vascular choroid		
					12. Eyestrain, headaches, and floaters		

### The global fungal outbreaks in COVID-19 patients

Although fungal disease outbreaks are rare, opportunistic fungi take advantage of the weakened immune system of COVID-19 patients [15, 31]. Geological differences have influenced the occurrences of fungal coinfection. Peng et al. [18] reported that the fungal coinfection rate was significantly higher in patients from Asia than non-Asian patients. With the uprising second wave of COVID-19, a rare fungal disease mucormycosis caused by *Mucor spp.* happened in India with a high mortality rate [32]. Though India dealt with the severity, other regions, including the USA, the UK, Australia, France, Brazil, and Mexico, also reported having black fungus cases [33]. On May 25, 2021, two black fungus cases in Dhaka, Bangladesh, were found in individuals recovered from COVID-19. In July and August 2021, another two patients aged 40 to 60 were also diagnosed with black fungus. They were at their post-recovery stage of COVID-19, and their second COVID-19 tests were also negative. Interestingly, one of them even received two doses of the COVID-19 vaccine [34]. Moreover, John et al. [31] have reviewed 41 case reports of COVID-19 and mucormycosis, where 29 were recorded from India. Until July 21, 2021, over 45,374 mucormycosis cases have been reported in India, whereas 4,322 have died [32, 35]. Symptoms of mucormycosis developed between 6 and 24 days from the onset of disease, and a six-day delay of treatment could lead to mortality up to 66% [36, 37]. Nevertheless, some individuals who did not have diabetes and took steroids were also diagnosed with mucormycosis, indicating that COVID-19 is a risk factor for mucormycosis [38].

Fungal coinfections, including 40 *Candida auris*-infected cases in the USA, *Candida glabrata*- and *Candida albicans*-associated cases in China, *Aspergillus flavus*- and *Aspergillus fumigatus*-related infections have also been documented in Europe [39]. In addition, between January and March 2020, 8 out of 104 COVID-19 patients infected with IPA have been found in China [40]. According to some other reports, the morbidity rate for IPA coinfection with COVID-19 patients was 20.6% in Belgium, 19.6% in the Netherlands, 33.3% in France, and 26% in Germany [41, 42]. A study found that when candidemia occurs with SARS-CoV-2, the mortality rate was 83.3%, even though the proper antifungal treatment was given to the patients [43]. In another study conducted in Colombia, 20 cases with around 30 days of observation while receiving antifungal therapy before achieving fungemia and taking up steroids due to COVID-19 came up with a 60% mortality rate [44]. Fekkar et al. conducted a study among 2723 hospitalized COVID-19 patients, whereas eight were positive for CAPA, while the morbidity rate was 0.03% for hospitalized individuals and 3.3% for ICU patients. Shockingly, all eight patients with CAPA were died [41]. Furthermore, an observational study on CAPA conducted by Nasir et al. in Pakistan found that the mortality rate was 44% [42].

### An overview of how fungi take the opportunity of the hampered immune system caused by SARS-CoV-2

SARS-CoV-2 anticipates the presence of angiotensin-converting enzyme-2 (ACE-2) receptor in the lung tissue, hence entering the lung cells with the help of furin. This entry site also provides virus stability [12]. The ACE-2 receptor has a downregulated expression in lung cells, leading to renin–angiotensin dysfunction in conjunction with acute lung injury. Followed by vascular leakage, inflammatory programmed cell death called pyroptosis stimulates inflammatory response locally. The result of pyroptosis is the secretion of different cytokines and chemokines in the blood, such as IL-1β, IL-6, IFN-γ, IFNγ-produced protein 10 (IP-10), and monocyte chemoattractant protein 1 (MCP1) [45]. SARS-CoV-2 has six ORFs in common with all coronaviruses, including ORF1a and ORF1b, which span more than two-thirds of the genome [46]. The ORF codes for nonstructural proteins (Nsps), accessory, and structural proteins. The papain-like protease (Nsp3), chymotrypsin-like, 3C-like or main protease (Nsp5), helicase (Nsp13), and RNA-dependent RNA polymerase (Nsp12) are believed to be involved in SARS-CoV-2 transcription and replication. Spike surface glycoprotein (S), membrane nucleocapsid protein (N), an envelope protein (E), and auxiliary proteins expressed by ORFs are four vital structural proteins in addition to Nsps [12]. While infected with SARS-CoV-2, Nsp3 of the virus leads to the cleavage of ISG15 from IRF3, therefore attenuating the type I IFN. Moreover, SARS-CoV-2 Nsp1 proteins suppress IFN responses. Regarding IRF3 nuclear translocation, SARS-CoV-2 ORF3b has a higher inhibitory impact [47].

Furthermore, SARS-CoV-2 ORF6 inhibits and prevents the generation of interferons (IFNs); consequently, the NF-κB (nuclear factor kappa-light-chain-enhancer of activated B cells) pathway becomes shut off [47]. Contrarily, when viral protein interacts with macrophages, it causes the production of cytokines such as IL-6, IL-10, IL-18, IL-12, IL1, and TNF-α [48]. The antigen-presenting cells (APC) present viral peptides to T-lymphocytes with the help of the MHC II complex (major histocompatibility complex class II), which conducts adaptive immune response by generating compromised T memory cells and releasing IFN-γ, IL-10, IL-17, and other chemokines subsequently. Survivors of COVID-19 are hence possessed with several cytokines and chemokines during infection called “cytokine storm” by dint of uncontrolled immune defense. Tocilizumab, infliximab, and serine protease inhibitors are applied to block the secretion of IL-6 and TNF-α and NF-κB expression to control hyper inflammation [49]. T cells and macrophages produce a smaller proportion of type II IFNs than natural killer cells. Type II IFNs induce apoptosis in infected cells and activate macrophages, natural killer cells, and T lymphocytes. Both type I and type II IFNs levels are decreased after in vitro stimulation of immune cells from COVID-19 patients is correlated with increasing disease extremity [50].

**Fig. 1:**
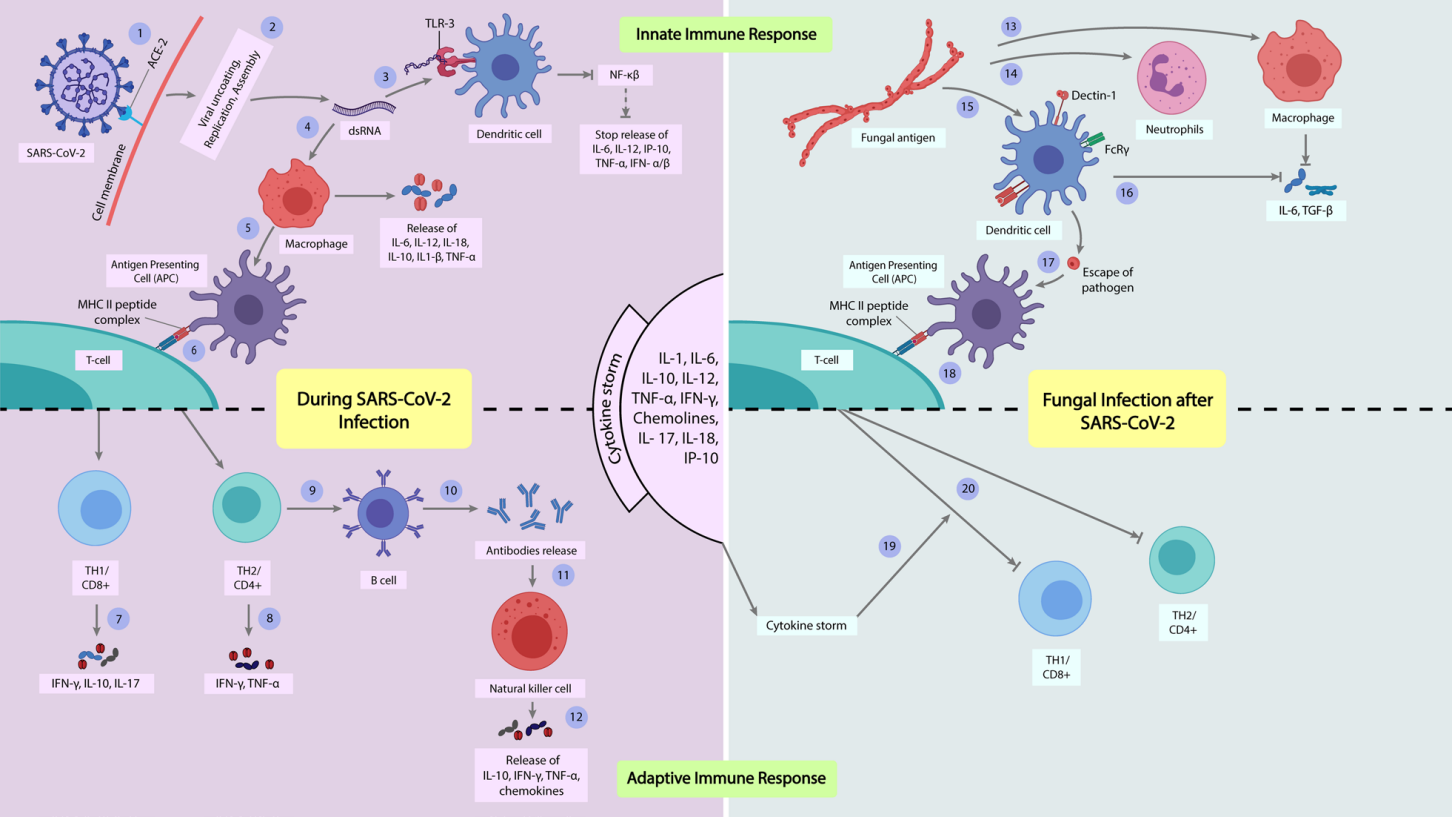
An overview of how fungi take the chances of the hampered immune system caused by SARS-CoV-2. ***Innate immune response in SARS-CoV-2 infection:*** SARS-CoV-2 enter the host cell through the attachment of viral spike (S) protein to ACE-2 (angiotensin-converting enzyme-2) receptor on the cell membrane **(1)**. Then ssRNA virus is reassembled to the dsRNA viral genome **(2)**. TLR-3 (Toll-like receptor-3) mutation stops the NF-κβ (nuclear factor kappa-light-chain-enhancer of activated B cells) pathway and prevents the release of cytokines and chemokines **(3)**. Macrophage releases IL-6, IL-10, IL-12, IL-18, IL1-β, and TNF-α **(4)**. Antigen-presenting cells (APC) present MHC II (major histocompatibility complex II) peptide complex on T cells **(5, 6)**. ***Adaptive immune response in SARS-CoV-2 infection***: TH1/CD8 + and TH2/CD4 + release IFN-γ, TNF-α, IL-10, and IL-17 **(7, 8)**. TH2 releases antibodies followed by activating B-cell **(9, 10)**. Antibodies activate NK (natural killer) cells to release cytokines and chemokines **(11, 12**). ***Innate immune response in fungal infection after SARS-CoV-2 infection***: Fungal complement protein stops the release of IL-6 and TGF-β **(13)**. Fungal pathogens activate neutrophils **(14)**. Pathogen attaches by dectin-1 in dendritic cells, and destructed immune system fails to release cytokines **(15, 16)**. Pathogen escapes and subsequently presents on APC (antigen-presenting cells) with the help of the MHC II peptide complex to activate T cells **(17, 18)**. ***Adaptive immune response in fungal infection after SARS-CoV-2 infection:*** Impaired adaptive immune response having cytokines from the “cytokine storm” results in the blockage of the proliferation of T cell into TH1/CD8 + and TH2/CD4 + **(19, 20)**

Fungal spores are first confronted by the first-line defense of the host, which subsequently results in an innate immune response. In conventional cases, fungal spores are engulfed by macrophages, killed by neutrophils, and attached to dendritic cells through receptor dectine-1. However, moving to the presentation of the fungal pathogen to APC, they faced IFN-γ or IL-17 (Th-17) that clear out them from the host cell. Host cells are embedded with many cytokines, mainly TNF-α, IL-1, and IL-6 [51]. Fungi are prone to be distinguished by the action of IFN-γ or IL-17 provided by T cells. Given impaired T cells and fewer other lymphocytes, fungi could not be eliminated from an immunosuppressed patient, especially if infected with SARS-CoV-2. To this extent, an opportunistic fungal coinfection in immunocompromised SARS-CoV-2 patients may result in short survival or cure [16] (Fig. 1).

### Control and prevention of fungal infections in COVID-19 patients

Prolonged hospitalization, long-time illness, lack of surveillance and early diagnosis, clinical mismanagement, and antibiotics that suppress the defense system of COVID-19 patients trigger the fungal coinfection [52]. For instance, bronchoscopy performed on COVID-19 patients is an approach of aerosol generation, which could affect immunocompromised patients with fungal spores. The detection of galactomannan (a polysaccharide antigen of *Aspergillus* spp. cell wall) from bronchoalveolar lavage fluid is quite a functional and prompt method to detect invasive aspergillosis in immunocompromised patients [53]. In addition, PCR tests could also be helpful in early diagnosis other than galactomannan tests [40]. For detection and control of the *Candida spp.*, screening could be performed regularly to determine its risk factors and revaluate treatment protocol routinely [54].

Voriconazole is considered a preliminary antifungal treatment that works effectively with amphotericin B deoxycholate. Isavuconazole is another antifungal drug that holds the same activity as voriconazole. Echinocandins with azole work rapidly against invasive aspergillosis. Several drugs are still under clinical trials, including the inositol acylase inhibitor fosmanogepix against invasive aspergillosis and oral triterpenoid beta-glucan inhibitor ibrexafungerp against invasive aspergillosis and candidiasis. Although there is no specific time limit for therapy for fungal coinfection, experts suggest taking the drugs for 6 to 12 weeks as a course [55].

## Conclusions

The severity of SARS-CoV-2 complexities rises with coinfection of fungi during or after SARS-CoV-2 infection. According to the assessment of fatality and number of illnesses, it may not be wrong to say that if pre-diagnosis does not happen or patients remain unchecked for fungal or other coinfection, another threat will emerge in the coming days. It has already been shown that mortality due to fungal coinfection does not change significantly, even if antifungal treatment is taking place to cure the disease. Pre-laboratory diagnosis should be given utmost attention to avoid a worsening condition. Several cautions should be maintained to limit the spread of fungal spores, including wearing masks, sanitizing, and maintaining cleanliness. Changing diet and acquiring the habit of sanitization could help to boost immunity.

## Data Availability

Not applicable.

## Abbreviations

SARS-CoV-2: Severe acute respiratory syndrome coronavirus-2
COVID-19: Coronavirus disease 2019
CNS: Central nervous system
ARDS: Acute respiratory distress syndrome
ICU: Intensive care units
Th: T helper cell
IL: Interleukin
CAPA: COVID-19-associated pulmonary aspergillosis
IPA: Invasive pulmonary aspergillosis
ACE-2: Angiotensin-converting enzyme-2
IFN: Interferon
IP-10: IFNγ-produced protein 10
MCP1: Monocyte chemoattractant protein 1
Nsps: Nonstructural proteins
ORF: Open reading frame
ISG15: Interferon-stimulated gene15
IRF3: Interferon regulatory factor 3
TNF: Tumor necrosis factor
APC: Antigen-presenting cells
MHC: Major histocompatibility complex
NF-κB: Nuclear factor kappa-light-chain-enhancer of activated B cells
